# Programmable diffractive deep neural networks enabled by integrated rewritable metasurfaces

**DOI:** 10.1038/s41598-025-19638-8

**Published:** 2025-10-13

**Authors:** Sanaz Zarei

**Affiliations:** Department of Electrical Engineering, Sharif University of Technology, Tehran, Iran

**Keywords:** On-chip programmable diffractive deep neural network, Phase-change metasurface, Sb_2_Se_3_, Direct laser writing, Machine learning, Integrated optics, Nanophotonics and plasmonics, Metamaterials, Photonic devices

## Abstract

Photonic neural networks capable of rapid programming are indispensable to realize many functionalities. Phase change technology can provide nonvolatile programmability in photonic neural networks. Integrating the direct laser writing technique with phase change material (PCM) can potentially enable programming and in-memory computing for on-chip photonic neural networks. Sb_2_Se_3_ is a newly introduced ultralow-loss phase change material with a large refractive index contrast over the telecommunication transmission band. Compact, low-loss, rewritable, and nonvolatile on-chip phase-change metasurfaces can be created by using direct laser writing on a Sb_2_Se_3_ thin film. Here, by cascading multiple layers of on-chip phase-change metasurfaces, an ultra-compact on-chip programmable diffractive deep neural network is theoretically demonstrated at the wavelength of 1.55 μm and benchmarked on two machine learning tasks of pattern recognition and MNIST (Modified National Institute of Standards and Technology) handwritten digits classification, and accuracies comparable to the state of the art are achieved.

## Introduction

 Recently, direct laser writing on Sb_2_Se_3_ phase-change thin films has been exploited as a simple, low-cost, and fast approach to create or reprogram photonic integrated circuits (PICs)^1^. Various photonic components consisting of waveguides, gratings, ring resonators, couplers, crossings, and interferometers were created in one writing step, without the need for additional fabrication processes^1^. Furthermore, writing imperfections (errors) can be easily corrected by locally adjusting each element using erasing and restoring^1^. Before this, Delany et al. employed the combination of large refractive index contrast and ultralow losses of Sb_2_Se_3_ and demonstrated the feasibility of a laser-programmed multiport router based on writing nonvolatile patterns of weakly scattering perturbations onto a multimode interference device^2^. Blundell et al. continued this work and could also establish the ability to strongly increase the effect per pixel/unit length by increasing the Sb_2_Se_3_ film thickness up to 100 nm in their very recent article^3^. Wu et al. took advantage of the direct laser writing of phase patterns optimized by the inverse design technique to achieve reconfigurability in programmable phase-change photonic devices^4^. They could demonstrate a photonic device that can be reconfigured from a wavelength-division demultiplexer to a mode-division demultiplexer^4^. Also, a rewritable PIC architecture based on selective laser writing on another phase change material (Sb_2_S_3_) has also been realized by Miller et al.^5^. Liu et al. reported a high-speed patterning approach using 780nm femtosecond laser pulses to accomplish partial re-amorphization of Sb_2_S_3_ to write and erase multiple microscale color images^6^. Their proposal can find many applications in non-volatile ultrathin displays.

Photonic neural networks (PNNs) may be the next-generation computing platforms due to their advantages of low power consumption, high parallelism, and light-speed signal processing. On-chip diffractive optical neural networks are one of many implementations of PNNs^7–9^, in which an on-chip high-contrast transmit array (HCTA) metasurface^10^ functions as an optical neural network hidden layer. Inspired by the previously presented on-chip diffractive optical neural networks^7–9^ and leveraging the technique of direct laser writing on Sb_2_Se_3_ thin film, a proof-of-principle of an on-chip programmable diffractive deep neural network is presented in this article for image classification.

## Phase-change metasurface

**Fig. 1:**
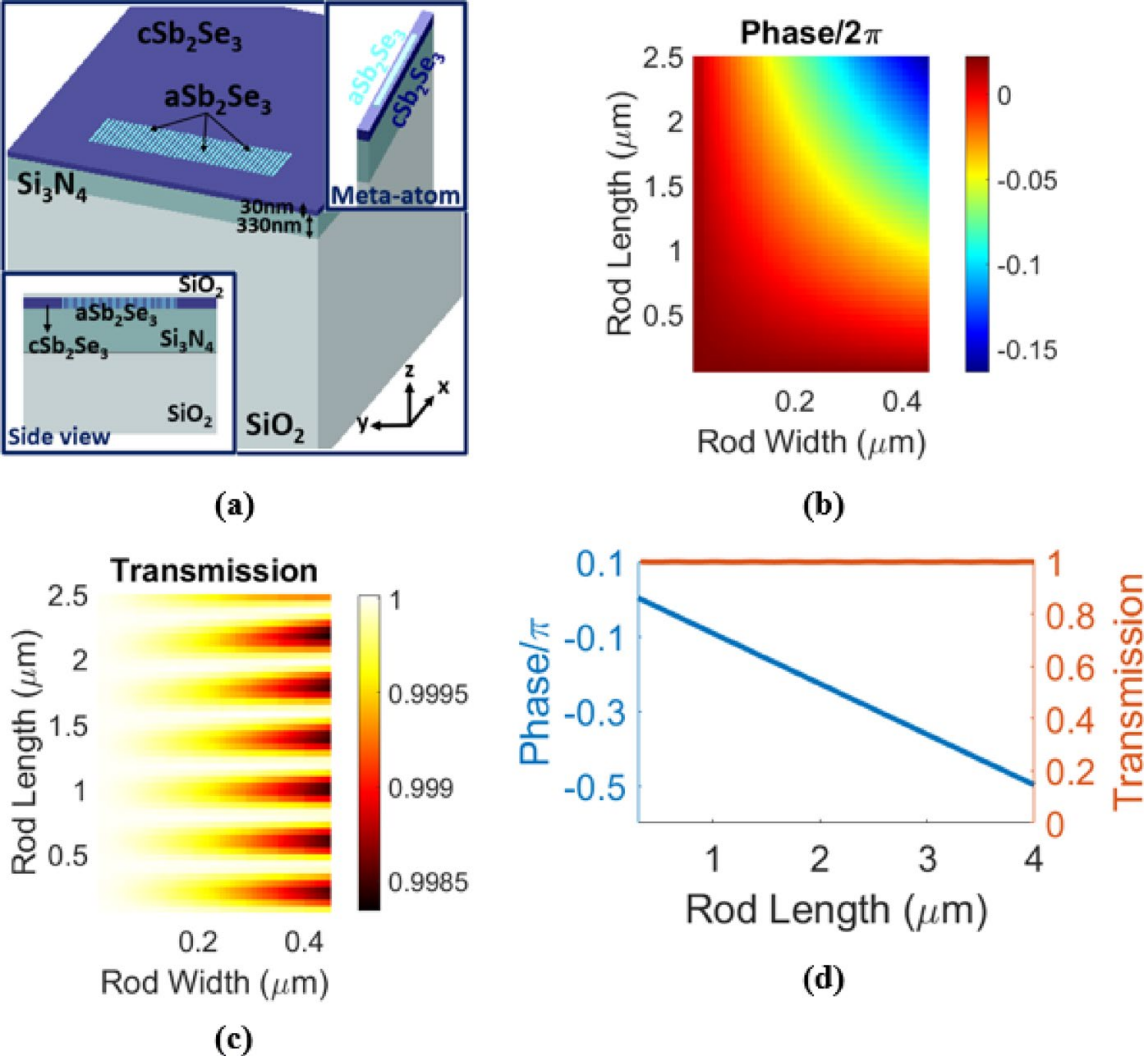
**(a)** Schematic of (aSb_2_Se_3_) rod array in the (cSb_2_Se_3_) thin film. The insets show the side view and a meta-atom of the structure. The electric field transmission phase **(b)** and amplitude **(c)** of the TE-polarized guided wave for a meta-atom versus the (aSb_2_Se_3_) rod length and width. **(d)** Electric field transmission phase and amplitude versus (aSb_2_Se_3_) rod length, fixing the rod width at 400 nm. These plots are generated by the commercial software package Lumerical FDTD. The operation wavelength is set to be 1.55 μm.

In this study, the on-chip programmable diffractive deep neural network consists of multiple phase-change metasurfaces, each of which performs as a neural network layer. The phase-change metasurface is a one-dimensional amorphous Sb_2_Se_3_ (aSb_2_Se_3_) rod array in the crystalline Sb_2_Se_3_ (cSb_2_Se_3_) thin film, as is shown in Fig. 1(a)^11^. Analogous to previous demonstrations^1–4^, the metasurface can be written (or rewritten) using direct laser writing. The lattice constant of the metasurface is 500 nm. The Sb_2_Se_3_ film is 30 nm thick, and it is coated with a SiO_2_ capping layer of 200 nm thickness^1^. The SiO_2_ layer is for protection and oxidation inhibition of the Sb_2_Se_3_ layer. Underneath the Sb_2_Se_3_, there is a Si_3_N_4_ film with 330 nm thickness on a standard oxidized silicon substrate^1^. A single neuron (or meta-atom) is formed by a single (aSb_2_Se_3_) rod. The geometrical parameters of an (aSb_2_Se_3_) rod can be learnable parameters. As is shown in Figs. 1(b) and 1(c), by adjusting the geometrical parameters of an (aSb_2_Se_3_) rod (i.e., the (aSb_2_Se_3_) rod length and width), the transmission phase shift and amplitude of a single neuron for TE-polarized guided wave can be tuned. By fixing the (aSb_2_Se_3_) rod width to 400 nm and altering the rod length from 300 nm to 4 μm, more than π/2 phase shift is attained, while the transmission amplitude is very near to 1 (Fig. 1(d)). Therefore, in the on-chip programmable diffractive deep neural network, the rod lengths of meta-atoms (neurons) are chosen as the learnable parameters, which collectively adjust the amplitude and phase profile of the output wavefront. The plots in Fig. 1 are generated using the commercial software package Lumerical FDTD. Throughout simulations, the Fundamental TE mode is selected for excitation, and the x-axis is chosen as the injection axis. The operation wavelength is set to be 1.55 μm.

## On-chip programmable diffractive deep neural network

Like artificial deep neural networks, an on-chip programmable diffractive deep neural network is comprised of one input layer, multiple hidden layers, and one output layer. Each hidden layer of the network is a phase-change metasurface that consists of many meta-atoms (neurons) arranged linearly. Each meta-atom is a weight element that connects to the meta-atoms on adjacent layers through diffraction and interference of light^7^. The input images are preprocessed and converted to a vector, and then encoded into the amplitude of the input light at the input layer. The output layer is composed of multiple detection regions aligned in a linear configuration. The network is trained on the training dataset using an error-backpropagation algorithm based on the adjoint gradient method described in^12,13^. For error-backpropagation, Adam optimizer is adapted to perform mini-batch gradient descent^14^. A decaying learning rate with a decay rate of 0.99 in each epoch is applied in the training process. A cost function in terms of the squared errors between the desired set of output intensity distributions (targets) and realized set of output intensity distributions is defined and minimized iteratively by adjusting the design parameters (i.e., (aSb_2_Se_3_) rod lengths through the metasurfaces). In the adjoint method, the gradient of the cost function with respect to all design variables can be computed using only two full-field simulations^12^. After numerical training, the inference performance of the designed network is numerically tested using the test dataset and subsequently verified using the 2.5D variational FDTD solver of the Lumerical Mode Solution. In verification, the preprocessed input images are encoded as the intensity of input light in Lumerical Mode Solution slab plane wave sources. Here, the capability of the on-chip programmable diffractive deep neural network is benchmarked on two machine learning tasks of pattern recognition for English letters X, Y, and Z and MNIST (Modified National Institute of Standards and Technology) handwritten (0-1-2) digits classification^15^.

### Pattern recognition

**Fig. 2:**
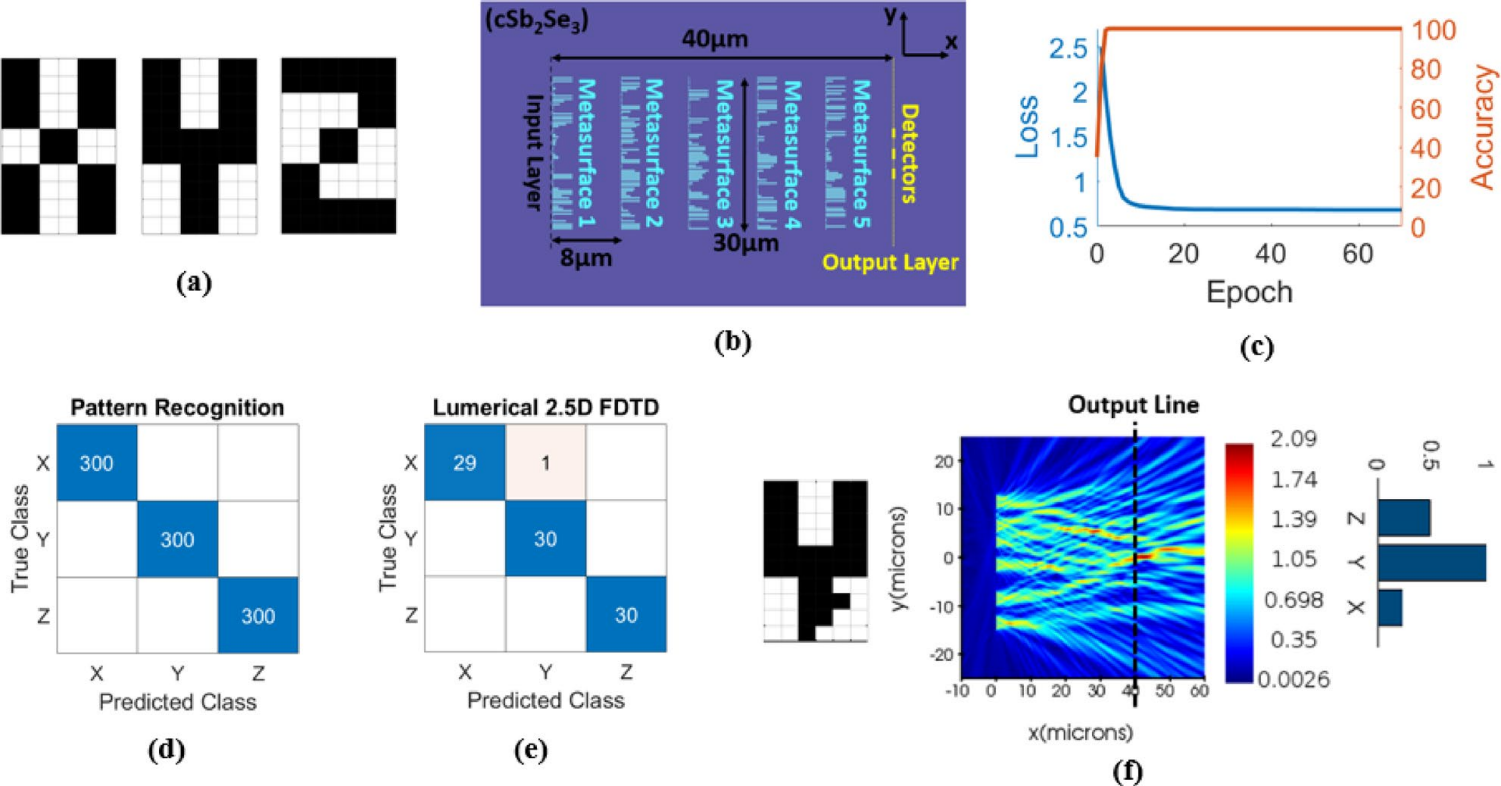
**(a)** 10 × 6-pixel images of the letters X, Y and Z, **(b)** 2D schematic of the five-layer network trained to perform as an on-chip pattern recognizer, **(c)** loss and training accuracy versus epoch during the learning process, **(d)** the confusion matrix of the network over 900 images of the test dataset, **(e)** the confusion matrix of the network, computed by 2.5D FDTD of Lumerical Mode Solution, over 90 random images from the test dataset, **(f)** the x-y view of the electric field distribution at the middle plane of the Sb_2_Se_3_ thin film through the whole network and the normalized power of the three output detectors for a representative Y image.

First, the on-chip programmable diffractive deep neural network is trained for pattern recognition of English letters X, Y, and Z. The input letters are the binary letter images with 60 pixels (Fig. 2(a)). The dataset is created by amplitude flipping in random single-pixel and double-pixels in the binary letter images of X, Y, and Z^7^. This generates 5490 images, of which 4590 images are used as the training dataset and fed through the metasystem in batches of 10 during each training epoch, and the remaining 900 images are used as the test dataset. The input patterns are reshaped from a two-dimensional 10 × 6 matrix to a 60-component one-dimensional vector^7^ and then encoded as the amplitude of the input light.

The pattern recognizer is composed of five phase-change metasurfaces, each of which consists of 60 meta-atoms (Fig. 2(b)). The 30 μm-length metasurfaces are precisely aligned with 8 μm separations. After light exits the fifth metasurface, it propagates 8 μm until it reaches the network output layer with three linearly arranged detectors (corresponding to the letters X, Y, and Z). The weights ((aSb_2_Se_3_) rod lengths) are initialized randomly in the range [300 nm, 4 μm] and kept in the same range during the training. Figure 2(c) illustrates the evolution of the loss and accuracy for the training set during the learning procedure. Only after 3 epochs, the metasystem can achieve 100% accuracy in English letter recognition. The blind testing accuracy of the designed metasystem is 100% over the test dataset. Verification with Lumerical 2.5D FDTD also indicates 98.8% matching with numerical testing results. For verification, 90 random patterns, 30 random patterns per letter, are selected from the test dataset. The confusion matrices for numerical testing and FDTD testing are shown in Figs. 2(d) and 2(e), respectively. The x-y view of electric field distribution in the network and the normalized power of three output detectors for a sample input pattern of letter Y are presented in Fig. 2(f). Table 1 summarizes the characteristics of the designed pattern recognizer.

### MNIST handwritten digits classification

For digit classification, the training is performed using 18,623 images of handwritten digits (0-1-2) from the MNIST handwritten digits database. The training images are fed through the network in batches of 64. After the training phase, the designed network is tested on 3147 images of the test dataset. The grayscale 28 × 28-pixel images are down-sampled to 14 × 14 pixels and converted to 196-component vectors. The vectors are then encoded as the amplitude of the input light.

**Fig. 3:**
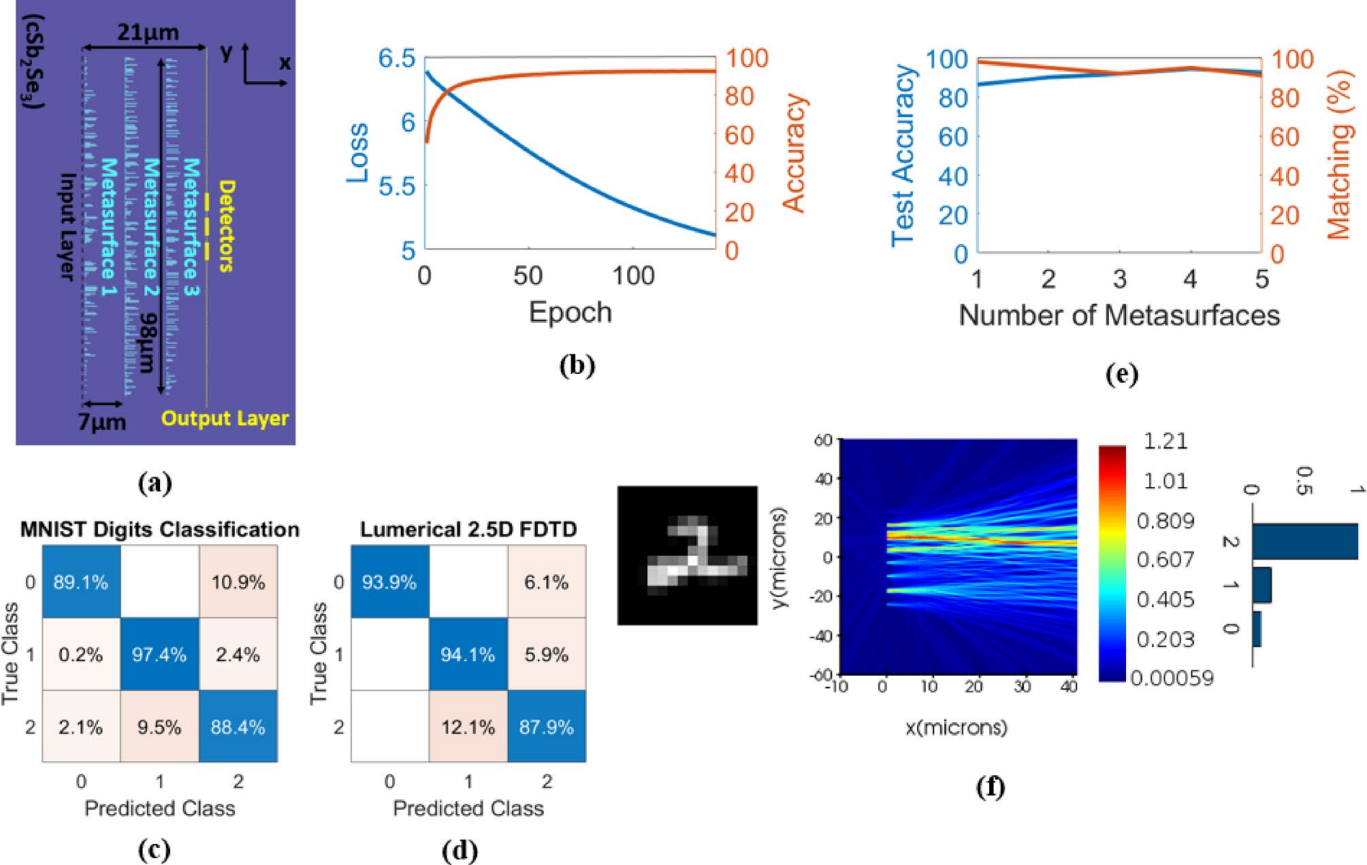
**(a)** 2D schematic of the three-layer network trained to perform MNIST (0-1-2) handwritten digits classification, **(b)** loss and training accuracy versus epoch during the learning process, **(c)** the confusion matrix of the metasystem over 3147 images of the test dataset, **(d)** the confusion matrix of the metasystem, calculated by 2.5D FDTD of Lumerical Mode Solution, over 100 random images from the test dataset, **(e)** blind testing accuracy for metasystems with different number of metasurfaces and the associated matching percentage between numerical testing and Lumerical 2.5D FDTD verification over 100 random images from the test dataset, **(f)** the x-y view of the electric field distribution at the middle plane of the Sb_2_Se_3_ thin film through the metasystem and the normalized power of three output detectors for a representative image of the digit 2.

The schematic of the digit classifier is illustrated in Fig. 3(a). It is composed of three phase-change metasurfaces, each with 196 meta-atoms. The length of the one-dimensional metasurfaces is 98 μm, and they are precisely aligned with 7 μm separations. After light exits the third metasurface, it propagates 7 μm until it reaches the output layer of the network with three linearly aligned detectors (corresponding to the digits 0, 1, and 2). The (aSb_2_Se_3_) rod lengths are initialized randomly and kept in the range of [300 nm, 5 μm] during the training. Figure 3(b) depicts the loss and training accuracy in each epoch during the learning process. After 140 epochs, the training accuracy reaches 92.38% over the whole training dataset. The blind testing accuracy of the finally designed metasystem is 91.86% over the test dataset. From the test dataset images that the designed network can successfully classify, 100 handwritten digit images are randomly selected for 2.5D FDTD verification. Verifications indicate that for 92 out of 100 images, similar predictions to numerical predictions are made, which means 92% matching between the two predictions. The confusion matrices for numerical testing and FDTD testing of the network are shown in Figs. 3(c) and 3(d), respectively. Also, the dependence of the digit classifier performance on the number of metasurfaces (hidden layers) is investigated in Fig. 3(e). As can be observed, the network blind testing accuracy grows from 86.30% for the one-layer network to 94.43% for the four-layer network and then declines to 92.50% for the five-layer network. The matching percentage between numerical testing and FDTD testing is above 90% for all the neural networks. The one-layer network has the best matching score of 98%, and the five-layer network has the worst matching score of 91%. Due to the nearly similar performance of all the networks in Fig. 3(e), the structural complexity and footprint may be the main determining factors. Figure 3(f) shows the x-y view of the electric field distribution in the three-layer metasystem and the normalized power of three output detectors for a sample image of the handwritten digit 2. The summarized characteristics of the presented image classifier are given in Table 1.

**Table 1:** Characteristics summary of the trained metasystems.

Characteristics	Pattern Recognizer	(0-1-2) Digit Classifier
Number of metasurfaces	5	3
Number of meta-atoms per metasurface	60	196
Number of design variables	300	588
Length of each on-chip metasurface	30 μm	98 μm
Distance between the input layer and the first metasurface	0	0
Distance between two neighboring metasurfaces	8 μm	7 μm
Distance between the last metasurface and the output layer	8 μm	7 μm
Total device size	30 μm×40 μm	98 μm×21 μm
Number of detectors	3	3
Length of each detector	2 μm	5 μm
Distance between two neighboring detectors	2 μm	2 μm
Detectors arrangement	Linear	Linear
Blind testing accuracy on the test dataset	100%	91.86%
Matching between numerical predictions and Lumerical 2.5D FDTD verifications	98.8%	92%

**Table 2:** Comparison between the previously presented On-chip diffractive optical neural networks and the presented scheme.

Ref	Platform	Phase Shifters	Programmable (Nonvolatile)?	Task	Number of Output Classes	Number of Layers	Number of Neurons per Layer	Footprint	Operation Wavelength	Operation Bandwidth
^7^	SOI	Air (SiO_2_) Slots	No	X-Y-Z Pattern Recognition	Three	Two	450 (Double slots)	450 μm× 300 μm	1.55 μm	1.53–1.625 μm
^8^	SOI	Air (SiO_2_) Slots	No	IRIS Flower Classification	Three	Three	186 (Triple slots)	280 μm× 1000 μm	1.55 μm	-
^16^	SOI	Thermo-optic	Yes (No)	Multimodal (vision, audio, touch)	Four	Five	16	0.36 mm× 1.42 mm	-	-
This work	(cSb_2_Se_3_) over Si_3_N_4_-O-I	(aSb_2_Se_3_) rods	Yes (Yes)	MNIST (0-1-2) digits classification	Three	Three	196	98 μm× 21 μm	1.55 μm	1.5–1.6 μm (100 nm)

## Discussion

### Performance

According to the numerical results, a five-layer English letters X, Y, and Z pattern recognizer and a three-layer (0-1-2) MNIST handwritten digits classifier based on the presented on-chip programmable diffractive deep neural network architecture could achieve 100% and 91.86% prediction accuracies on the test dataset, respectively. FDTD testing of these structures reveals 98.8% and 92% matching with numerical testing results, respectively. Compared to the three-class pattern recognizer presented in^7^ for binary letter images with 15 pixels, which has numerical and FDTD testing accuracy of 98% and 95.8%, respectively, and a footprint of 450 μm×300 μm, the presented scheme has a much smaller footprint of 30 μm×40 μm for the 60-pixel pattern recognizer and 98 μm×21 μm for the more complex digits classifier, while showing comparable accuracies. It is also worth comparing our proposal to the three-class Iris flower classifier presented in^8^ with a footprint of 280 μm×1000 μm that has a numerical and experimental testing accuracy of 90% on the test dataset. Furthermore, both of those proposals^7,8^ are not programmable, and once fabricated, their Function is fixed and cannot be retrained for other Functions. A programmable multimodal scheme with 85.7% accuracy on multimodal (vision, audio, touch) test sets was previously presented in^16^, however, its footprint is very large (0.36 mm×1.42 mm). Moreover, the tunable diffractive units are implemented with thermo-optic phase shifters that are large-sized (3 μm×100 μm) and nonvolatile (which need a constant power supply to operate). Table 2 summarizes the characteristics of the previously presented on-chip diffractive optical neural networks and provides a comparison to the presented scheme in this article.

In the previous works based on direct-laser writing on Sb_2_Se_3_ thin film^2,3^, nonvolatile pixel perturbation patterns on a multimode interference device were optimized using an iterative method. Wu et al. employed a topology optimization approach to inverse-design the phase pattern of phase-change (Sb_2_Se_3_) multimode interference devices in a pixel-wise manner^4^. Adjoint optimization of on-chip phase-change (Sb_2_Se_3_) metasurfaces is a fast and efficient alternative optimization approach to those presented in^2–4^ to design phase change photonic devices. Furthermore, more complicated photonic devices with larger device areas can be designed. Other lithography-free reconfigurable demonstrations of on-chip photonic devices like photonic neural networks based on optical coding of spatial-temporal modulations of the imaginary index on an active III–V semiconductor platform^17^ and multimode interference power splitters based on plasma dispersion in planar silicon from all-optical excitation with spatially modulated pump beams^18,19^, although providing a convenient and cost-efficient paradigm for programmable integrated photonic circuits, suffer from a volatile nature. An on-chip programmable convolutional neural network based on a phase-change metasurface mode converter was also reported in^20^ by using phase-change material Ge_2_Sb_2_Te_5_ (GST). A photonic kernel with a 2 × 2 array of GST converters was experimentally demonstrated to perform MNIST handwritten (1–2) digits classification, which achieved experimental accuracy of 91% (91 out of 100 cases). However, the footprint of each GST converter is 80 μm×20 μm. Furthermore, the weight parameters are represented by 64 distinguishable levels created by partial phase transition of GST, which is rather difficult to implement.

The computational scale of many ONN architectures like integrated Mach-Zehnder interferometer grids, micro-ring modulator arrays, etc., is typically limited to 4 × 4 matrix-vector multiplications or smaller^16^. Therefore, most existing ONNs still struggle with classic tasks and small datasets like MNIST and Fashion-MNIST^16^. The on-chip diffractive optical neural networks, however, are supposed to enable large computational scales in the literature^8,16^. Our investigations indicate that these networks also are able to classify only 3–4 classes, suggesting fundamental limitations in their computational scale^21^. If the presented scheme in this article is benchmarked on the images of ten (0–9) handwritten digits from the MNIST dataset (for which the distance between two neighboring metasurfaces is 17 μm, the distance between the last metasurface and the output layer is 57 μm, the total device size is 98 μm×91 μm, and all other characteristics are similar to the ones presented in Table 1) the test accuracy and the FDTD matching score will be 76.02% and 30%, respectively. Therefore, Full 10-digit MNIST classification necessitates integration of 3 to 4 computing modules, each of which classifying 3 digits. More elaboration on the proposed hypothesis was presented in^21^, the summary of which is by increasing the classification categories, the classification accuracy of the on-chip diffractive optical neural networks degrades dramatically (e.g. for this scheme, the testing accuracy decreases from 91.86% for three classes to 76.02% for ten classes and matching score decreases from 92% for three classes to 30% for ten classes). Therefore, for the presented scheme, it can be deduced that the FDTD testing accuracy for ten (0–9) digits classification is about %76.02×%30 = 22.8%, which is rather a low classification accuracy. This consequence is signified when, for such neural networks, increasing the number of layers and/or the number of neurons per layer and/or changing the distance between the layers, don’t make a big difference in the classification accuracy, especially the FDTD testing accuracy^21^. These denote the limited capability of such a scheme to perform complex (multi-class) tasks^21^. As is described in^21^, the confusion matrices of many MNIST and Fashion-MNIST benchmarks with different numbers of classes reveal that these networks are able to classify only 3–4 classes correctly.

In the presented study, angular spectrum wave propagation that exploits fast Fourier transforms (FFTs) for formulating the diffraction is utilized to numerically simulate the on-chip programmable diffractive deep neural networks^12,13^. The numerical simulation results are verified using the 2.5D variational FDTD solver of the Lumerical Mode Solution. Despite very close matching between numerical simulation results and FDTD simulation results, a small discrepancy exists between them. This discrepancy is mainly justified by the differences in propagation models and discretization between diffraction-based modeling methods (like angular spectrum method) and full-wave electromagnetic modeling methods like FDTD.

**Fig. 4:**
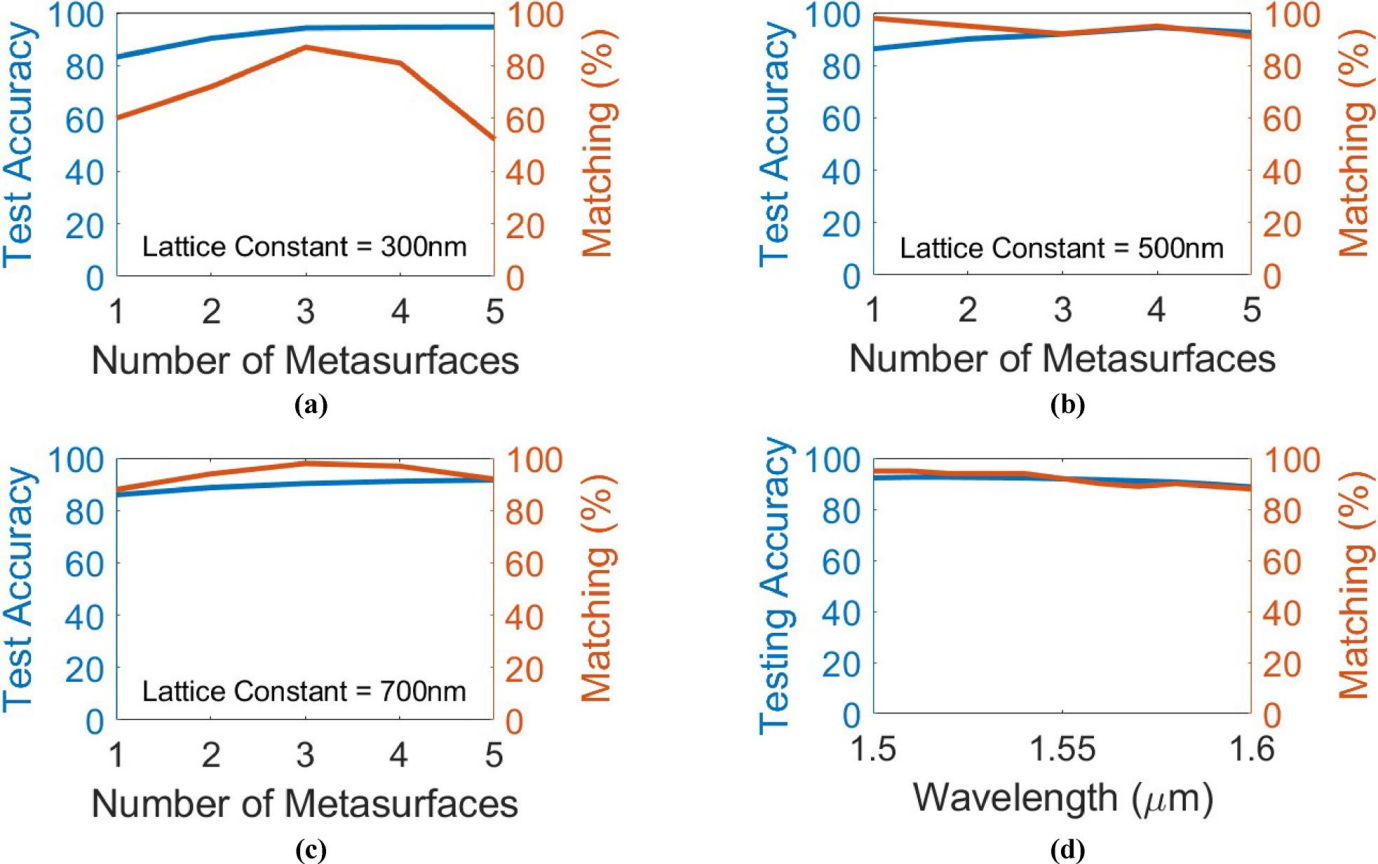
These figures illustrate how the subwavelength extent of phase-change metasurfaces affects the performance of the presented architecture. The blind testing accuracy for (0-1-2) MNIST handwritten digits classifier with different numbers of metasurfaces and the associated matching percentage between numerical testing and Lumerical 2.5D FDTD verification over 100 random images from the test dataset are shown for metasystems composed of **(a)** 300 nm-lattice constant phase-change metasurfaces, **(b)** 500 nm-lattice constant phase-change metasurfaces, and **(c)** 700 nm-lattice constant phase-change metasurfaces. **(d)** The blind testing accuracy and the matching percentage between numerical testing and Lumerical 2.5D FDTD verification over 100 random images from the test dataset, versus operation wavelength for the (0-1-2) MNIST handwritten digits classifier of Fig. 3.

The main challenge for the on-chip diffractive optical neural networks is the discrepancy between diffraction-based analysis methods and experimental/full-wave electromagnetic verifications. Many prior works^7–9,21–26^ tried to figure out the reason for this discrepancy. In this article, we add a few more analyses of such 2D structures to the former analyses. Figure 4 presents the results of these analyses. In Fig. 4, the performance of the (0-1-2) MNIST handwritten digits classifier with different numbers of metasurfaces (neural network layers) is investigated for different lattice constants of the phase-change metasurfaces. The lattice constant of the metasurfaces is set to 300 nm, 500 nm, and 700 nm in Figs. 4(a), 4(b), and 4(c), respectively. For these figures, the (aSb_2_Se_3_) rod width is fixed at 250 nm, 400 nm, and 600 nm, respectively, and the (aSb_2_Se_3_) rod length is selected as the learnable parameter. As is evident, while the testing accuracies are very near for the three lattice constants, the matching score between numerical and FDTD testing falls down by decreasing the lattice constant. Especially, for the more subwavelength 300 nm lattice constant (Fig. 4(a)), the matching score gets very low. In Fig. 4(d), the performance of the three-layer (0-1-2) MNIST handwritten digits classifier shown in Fig. 3 is studied when changing the operation wavelength. It is seen that the performance of the network remains nearly the same in the wavelength range of 1.5 μm to 1.6 μm, which indicates the broadband operation of the presented scheme. It should be mentioned that by increasing the wavelength, a slight decay in the testing accuracy as well as the matching score between the numerical and FDTD testing can be observed. This also indicates that a more subwavelength meta-atom results in a smaller matching score between numerical and FDTD testing. For the presented study in Fig. 4(d), the refractive index of Sb_2_Se_3_ between 1.5 μm and 1.6 μm is nearly constant^27^, and the refractive indices of silicon nitride and silicon oxide are extracted from^28,29^, respectively.

### Practical issues

For the presented on-chip optical neural network scheme, there might be a simulation-to-reality gap, as is also indicated in^8^ for the on-chip diffractive optical neural networks based on on-chip HCTA metasurfaces. This gap was attributed to the errors caused by the fabrication process in^8^, and an algorithm compensation method consisting of phase compensation and power compensation was exploited to reduce the negative impacts of errors. The phase compensation stage was implemented based on an online in-situ training procedure. Here, the rewritable nature of the on-chip optical neural network helps to easily perform the compensation stage. Furthermore, the whole training process of the on-chip optical neural network can be easily accomplished online and in situ by erasing and writing the diffractive units. In the context of on-chip diffractive optical neural networks, the presented programmable scheme can be a breakthrough, because it provides the opportunity to retrain these networks by in-situ training very easily. Direct laser writing technique makes it straightforward to adjust each neuron individually and therefore correcting the errors to restore the designed functionality^1^. Using the presented scheme and direct laser writing technique will assist finding the main reason for the limited scalability of these networks and if it is because of the incapability of Fourier-optics methods in modeling the evolution of optical fields through the network, then in-situ training would be an ideal solution for this problem.

In our study, (aSb_2_Se_3_) rod array in the (cSb_2_Se_3_) thin film is considered as the phase-change metasurface. According to^3^, the method of direct laser writing of amorphous rods on a crystalline background leads to small pixels with well-defined edges. However, this can be achieved at the cost of much increased losses, as most of the Sb_2_Se_3_ layer is crystallized. On the other hand, for (cSb_2_Se_3_) rod array in the (aSb_2_Se_3_) thin film, direct laser writing of crystalline rods on an amorphous background offers limited spatial control over crystalline rods, generally resulting in larger rod size compared with amorphous rods^3^. In^1^, a commercial laser writing system (Heidelberg DWL 66+, 405-nm laser) was utilized to directly write the circuit layouts on a blank (cSb_2_Se_3_) thin film. The minimum achievable feature size with such an industrialized laser writing tool was 300 nm. In this article, for the presented on-chip programmable optical neural network designs to be complied with commercial laser writing constraints, the width of (aSb_2_Se_3_) rods is fixed at 400 nm, and the minimum value for (aSb_2_Se_3_) rods lengths is set to be 300 nm in the training processes. The thickness of the Sb_2_Se_3_ film is assumed to be 30 nm. As is mentioned in^3^, switching depths exceeding 200 nm are achieved for direct-write amorphization pulses in (cSb_2_Se_3_) thin films. Therefore, it is expected that 30 nm thickness to be in the regime where the entire thickness of the Sb_2_Se_3_ is switched.

As is indicated in^3^, losses stay very low for increasing thickness and length of Sb_2_Se_3_ in the amorphous state (in a waveguide). However, generally losses increase exponentially with the length of Sb_2_Se_3_ in both amorphous and crystalline states. While in^3^, these losses are attributed to surface and volume contributions, because of scattering by sidewall surface roughness and small poly-crystalline domains, Wu et al. considered the scattering induced by the grain boundaries within the (cSb_2_Se_3_) waveguide as the main source of loss^1^. According to^1^, the scattering loss can be mitigated when a lower erasure temperature is used to form larger crystal grains with sizes on the order of tens of micrometers. Based on^3^, the insertion loss over the device length dramatically reduces by decreasing the film thickness, however, larger Sb_2_Se_3_ film thickness leads to increased modulation per Sb_2_Se_3_ film length. As a result, due to^1,3^, a 30 nm-thickness Sb_2_Se_3_ film will have a propagation loss between (0.003–0.01) dB/µm, which is rather negligible, considering the small propagation lengths in the presented schemes.

**Fig. 5:**
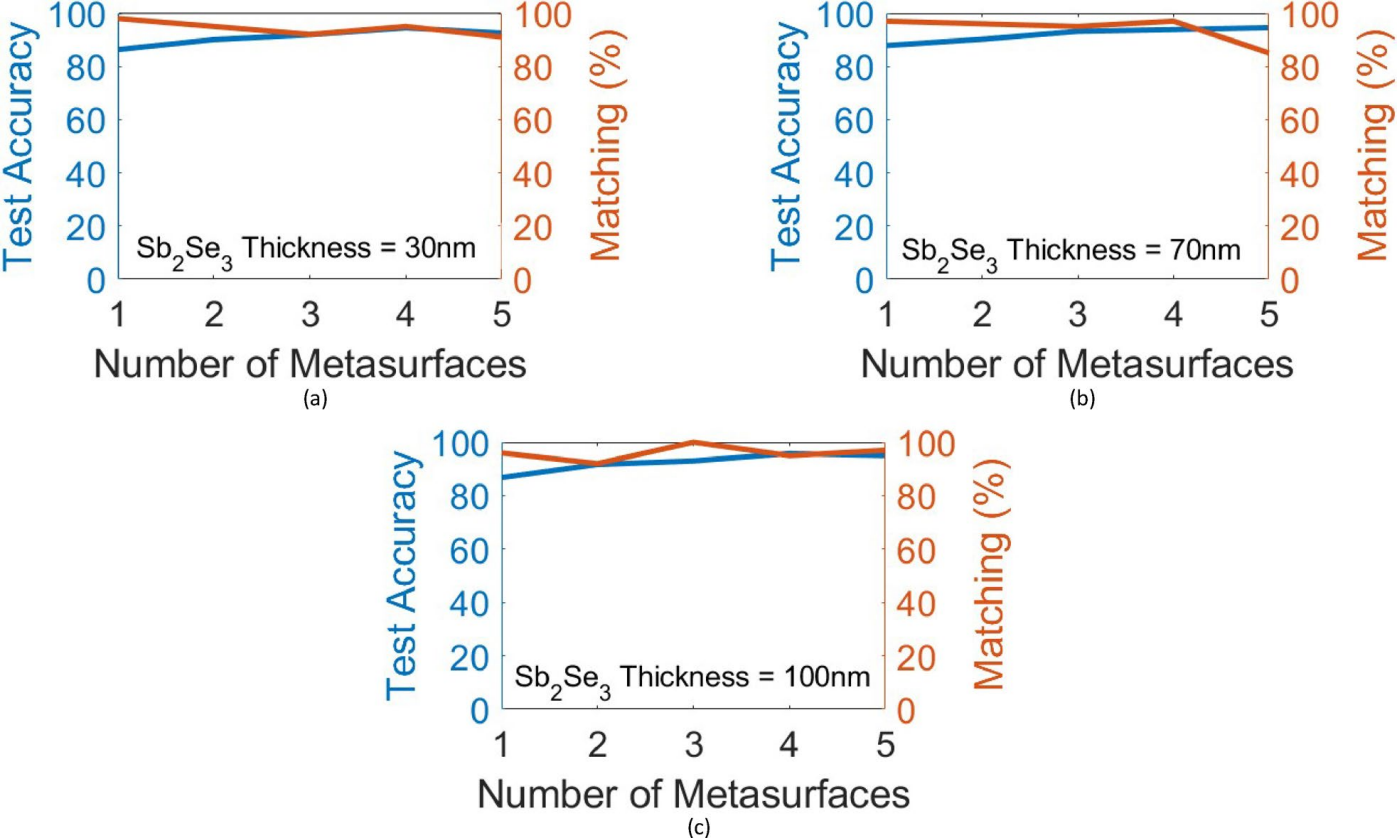
These figures illustrate how the phase-change film thickness affects the performance of the presented (0-1-2) MNIST handwritten digits classifier. The blind testing accuracy of the classifier with different numbers of metasurfaces and the associated matching percentage between numerical testing and Lumerical 2.5D FDTD verification over 100 random images from the test dataset are shown for (cSb_2_Se_3_) film thicknesses of **(a)** 30 nm, **(b)** 70 nm, and **(c)** 100 nm.

According to^3^, thicker Sb_2_Se_3_ layers can provide an increase in the induced optical phase shift of each neuron ((aSb_2_Se_3_) rod), however, this is achieved at the cost of increased losses by absorption, scattering, and multimode contributions. Figures 5(a), 5(b), and 5(c) show the performance of an MNIST (0-1-2) digit classifier with different numbers of metasurfaces (hidden layers) for the (cSb_2_Se_3_) film thicknesses of 30 nm, 70 nm and 100 nm, respectively. A slight improvement in the matching score between analytical testing and 2.5D FDTD testing is observed. For (cSb_2_Se_3_) film thicknesses of 70 nm and 100 nm, the (aSb_2_Se_3_) rods’ lengths vary in the range [300 nm, 2 μm], as this range provides more than π/2 phase shift for each neuron ((aSb_2_Se_3_) rod). The (cSb_2_Se_3_) film losses are not taken into account in these investigations because of the lack of approximate loss values for these thicknesses in previous studies^1,4^. The loss values presented in^3^ were measured for Sb_2_Se_3_ films with different thicknesses deposited on silicon (SOI platform), which differs from the Si_3_N_4_-on-insulator platform used in this study. However, the losses are expected to be negligible due to the small propagation distance of 21 μm in this network.

Also, it can be deduced from Fig. 5 that increasing the number of metasurfaces in the network generally results in a higher numerical accuracy. However, while the matching score exists very high for most of the instances, its variations seem to follow a random trend with increasing the number of layers. Therefore, it can be concluded that increasing the number of metasurfaces slightly improves the performance for this scheme. This trend was also observed for X-Y-Z letter recognition in the related investigations. But it depends on the task and cannot be generalized to all tasks.

**Fig. 6:**
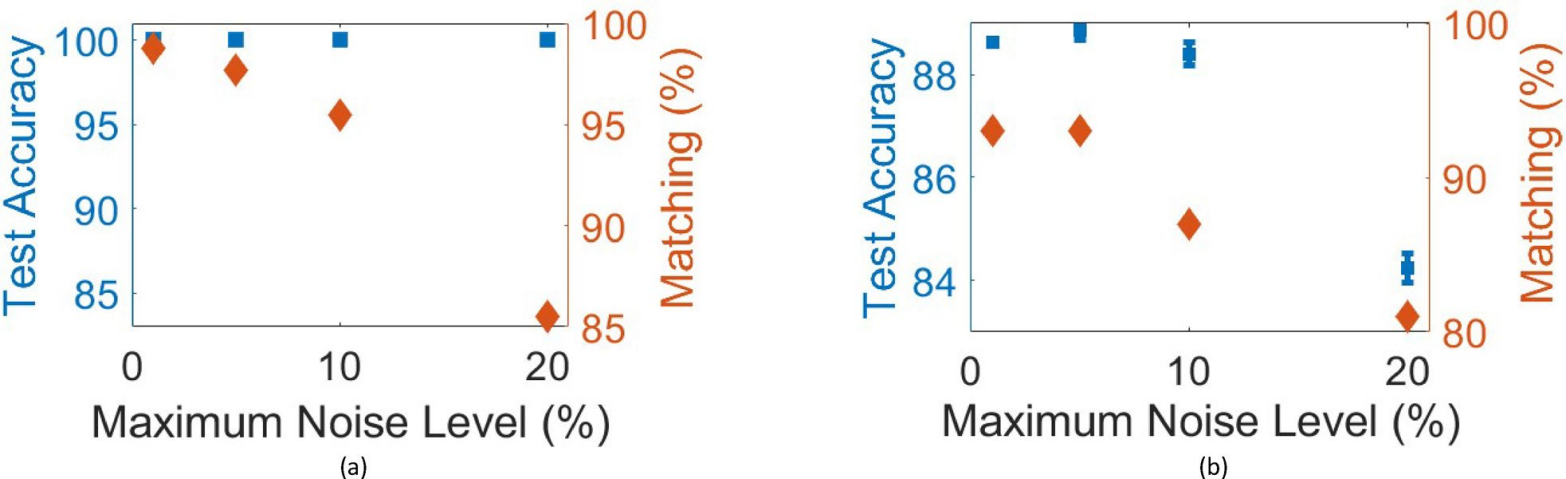
These figures illustrate how noisy input affects the performance of the presented scheme trained for **(a)** X-Y-Z pattern recognition, **(b)** (0-1-2) MNIST handwritten digits classification. The blue markers indicate the blind testing accuracy of the network over the test dataset, and the red markers indicate the matching score between numerical testing and Lumerical 2.5D FDTD verification over 90 and 100 random images from the test dataset for X-Y-Z pattern recognition and (0-1-2) MNIST handwritten digits classification, respectively. The error bars in (b) show the results for three testing experiments.

According to maximum propagation loss (4.5 dB/mm) that was mentioned in^1^ for photonic integrated circuits, the simulations are repeated with (cSb_2_Se_3_) extinction coefficient equal to $k=2.5561 \times {10^{ - 4}}$. The matching score between numerical testing and FDTD testing remains the same as the system without loss for X-Y-Z pattern recognizer (98.8%) and MNIST (0-1-2) digits classifier (92%). Also, the performance is investigated for the extinction ratio of $k={10^{ - 3}}$ that is equivalent to an approximate propagation loss of 17.6 dB/mm, and the performance remains the same for both X-Y-Z pattern recognizer and MNIST (0-1-2) digits classifier.

It should be mentioned that the presented scheme is mainly applicable to reprogrammable devices that don’t necessarily require very fast switching, and a Sb_2_Se_3_ endurance of thousands to millions of cycles is sufficient for them^2,3^. Further improvements in the surrounding materials’ thermal design and integration into highly optimized device configurations can substantially improve switching speed and endurance^2,3^.

Finally, our simulations show that the system is able to tolerate noisy or imperfect input encoding. Figure 6 shows the results of these simulations. In the simulations, random noise is applied to the input images. The applied maximum noise levels are [1%, 5%, 10%, 20%] of the maximum signal level. The testing accuracy on the test set remains 100% for all noise levels in X-Y-Z pattern recognition, and the matching score modifies to [98.8%, 97.7%, 95.5%, 85.5%] (Fig. 6(a)). The testing accuracy on the test dataset drops from 91.86% to [88.62%, 88.87%, 88.40%, 84.23%] for MNIST (0-1-2) dataset (Fig. 6(b)). The error bars show the results for three testing experiments. The matching score changes from 92% to [93%, 93%, 87%, 81%].

## Conclusion

In conclusion, by applying the direct laser writing technique on Sb_2_Se_3_ phase change material to direct-write or rewrite phase-change metasurfaces, an ultra-compact on-chip programmable diffractive deep neural network can be created. This technique provides a simple, low-cost, and fast approach to reprogram diffractive deep neural networks. With this reconfigurable and nonvolatile metasystem, the feasibility of a variety of functionalities such as optical computing and machine learning tasks can be explored more rapidly. In this article, numerical testing and FDTD testing results for two machine learning tasks of three-letter pattern recognition and more complicated three-handwritten-digits (from the MNIST dataset) classification imply that the network can properly perform these tasks.

## Data Availability

The datasets generated during and/or analyzed during the current study are available from the corresponding author on reasonable request.
